# Raman and Terahertz Spectroscopy of Low-Frequency Chiral Phonons in Amino Acids

**DOI:** 10.1021/acs.nanolett.6c00060

**Published:** 2026-04-20

**Authors:** Rahul Rao, Wonjin Choi, Joseph M. Slocik, Thuc T. Mai, Michael A. Susner, Kelsey A. Collins, Michael J. Newburger, Petr Bouř, Nicholas A. Kotov

**Affiliations:** † Materials and Manufacturing Directorate, Air Force Research Laboratory, Wright-Patterson AFB, Ohio 45433, United States; ‡ Physical and Life Sciences, Lawrence Livermore National Laboratory, Livermore, California 94550, United States; § BlueHalo, an AV company, Dayton, Ohio 45433, United States; ⊥ Core4ce, Dayton, Ohio 45422, United States; ∥ Institute of Organic Chemistry and Biochemistry, Flemingovo nám. 2, 16610 Prague, Czech Republic; ¶ Department of Chemical Engineering, 1259 University of Michigan, Ann Arbor, Michigan 48109, United States; # Biointerfaces Institute, University of Michigan, Ann Arbor, Michigan 48109, United States; ∇ Department of Materials Science and Engineering, University of Michigan, Ann Arbor, Michigan 48109, United States; ○ Center for Complex Particle Systems, University of Michigan, Ann Arbor, Michigan 48109, United States

**Keywords:** chiral phonons, Raman optical activity, terahertz spectroscopy, terahertz circular dichroism (TCD), amino acids, collective motions

## Abstract

Chiral phonons are mirror-symmetric vibrations with nonzero angular momenta that correspond to twisting and rotational motions of multiple atoms. In chiral crystals, these include low-energy terahertz (THz)-range vibrations of the molecular segments involving dozens of atoms with energies sensitive to molecular chirality. Here, we present spectral signatures of chiral phonons in circularly polarized Raman optical activity (ROA) spectra from enantiomers of amino acid crystals. Along with complementary THz circular dichroism (TCD) measurements, our ROA data reveal several vibrational bands in enantiomers of valine, alanine, tyrosine, and proline between 30 and 150 cm^–1^ (∼1–4.5 THz) that exhibit opposite intensities. Density functional theory calculations confirm their assignment to twisting and shearing molecular motions. The simultaneous registration of chiral phonon modes by ROA and TCD demonstrates the necessity of these complementary techniques to identify complex mirror-asymmetric vibrational modes and offers new insights into their interactions with circularly polarized light.

## Graphical abstract

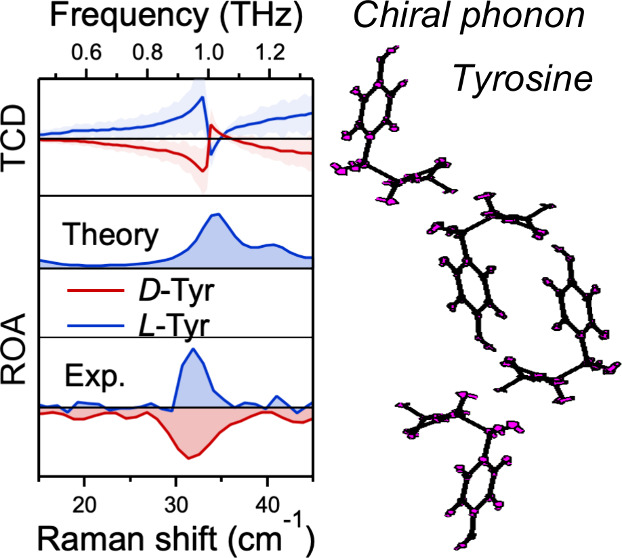

Chiral objects, i.e., those that cannot be geometrically superimposed on their mirror image,^1^ give rise to structural asymmetry between right- and left-handed enantiomers of molecules, nanoparticles, their assemblies, and other chemical structures. In the case of crystals, chirality is also associated with phonons, which are the propagating collective vibrational motions of atoms in the lattice. While phonons have traditionally been considered to possess only linear momentum, recent experimental and theoretical observations in both magnetic and nonmagnetic materials have revealed the existence of chiral phonons, which carry nonzero (pseudo)­angular momentum (PAM).^2–9^ Chiral phonons can be defined as quantized vibrational modes in which atoms or groups of atoms in a solid undergo rotational motion perpendicular to the direction of propagation of the vibration within the lattice. They have been observed using various spectroscopic techniques in enantiomers of both organic and inorganic materials^6,10–13^ and as transient phenomena in achiral two-dimensional (2D) lattices.^5,14,15^ Their growing significance makes them important for diverse applications^16^ and properties including ferroelectricity,^17^ topological properties,^18^ photovoltaics,^19^ spintronics,^20,21^ and phononics.^22^

There are two primary ways to register chiral phonons. First, slight differences in the angular momenta of the incident and scattered phonons result in a small but finite (typically a few cm^−1^) splitting in peak frequencies when excited by right- or left-handed circularly polarized (RCP and LCP, respectively) light.^16^ The second approach is to measure optical activity, which is the ability of chiral phonons to interact differently with circularly polarized light. Two typical techniques for such measurements are vibrational circular dichroism (VCD) and Raman optical activity (ROA), which are chiroptical extensions of infrared (IR) absorption spectroscopy and Raman scattering, respectively. VCD relies on the differential absorption of RCP and LCP light (*I*_R_ – *I*_L_) during a vibrational transition, while ROA measures the differential intensity of Raman scattered light for RCP and LCP excitation or detection.^23–26^ Lately, IR measurements in the terahertz (THz) frequency range, i.e., terahertz circular dichroism (TCD) and terahertz optical rotation dispersion (TORD), were added to this line-up as the most promising new methodologies to identify chiral phonons in crystals made from organic molecules^6^ rather than inorganic 2D lattices. Besides the areas of applications mentioned above, organic and biological materials with chiral phonons offer a wide range of structural and spectral tunability and solution-state processability.

Typical ROA measurements on organic materials have been limited to the high-frequency chemical fingerprint region (800–2000 cm^–1^), where the modes correspond to bending and stretching (localized motions) of organic moieties. In contrast, chiral phonons generally correspond to longer-range, lower-energy vibrations of the crystal lattices assembled from chiral molecules. These modes occur in the low-frequency regions in the THz part of the spectrum, necessitating the transition from VCD instrumentation operating in the mid-IR part of the spectrum to TCD instrumentation in the THz range.^10,27,28^ Recent studies on chiral phonons in multiple chemical structures, including nanostructured microparticles, crystalline nanowires, and microscale crystals, have revealed low-frequency chiral phonons between 1 and 2 THz,^10,28^ and although their ROA response might be anticipated, it has not yet been investigated through direct comparison with TCD. While the transitions sampled by the absorption of a THz beam and Raman scattering of visible laser light are similar, their intensities differ due to the distinct selection rules governing IR and Raman spectroscopy;^29^ thus, understanding these variations would be highly significant. Here, we take advantage of the overlap in energies/frequencies between TCD and low-frequency Raman spectroscopy (0–4.5 THz or 0–150 cm^–1^) and present TCD and ROA spectra from low-frequency chiral phonons in enantiomers of amino acid (AA) crystals such as valine, alanine, tyrosine, proline, and glutamine.

We begin with a comparison of THz absorption (TA)/TCD and Raman/ROA spectra from enantiomers of three AA exemplars, valine, alanine, and tyrosine; chemical structures of *D*- and *L*-Val, *D*- and *L*-Ala, and *D*- and *L*-Tyr are shown in parts a–c of Figure 1, respectively. Valine crystallizes in a monoclinic (*P*2_1_ space group) crystal structure, while alanine and tyrosine crystallize in orthorhombic (*P*2_1_2_1_2_1_ space group) crystal structures (Tables S1 and S2 detail our single-crystal X-ray diffraction refinements for *L*-Val and *L*-Ala, respectively). *P*2_1_ and *P*2_1_2_1_2_1_ correspond to the two main space groups that amino acids typically crystallize in and contain 1 and 3 screw axes, respectively. Importantly, the crystal lattices of alanine, valine, and tyrosine have 52, 76, and 96 atoms/unit cell, which leads to 156, 228, and 288 total normal modes for these crystals, respectively. Tyrosine also has α and β polymorphs—slightly different solid-state forms—which further increases the number of possible vibrational modes that can be potentially observed in one sample.

**Figure 1:**
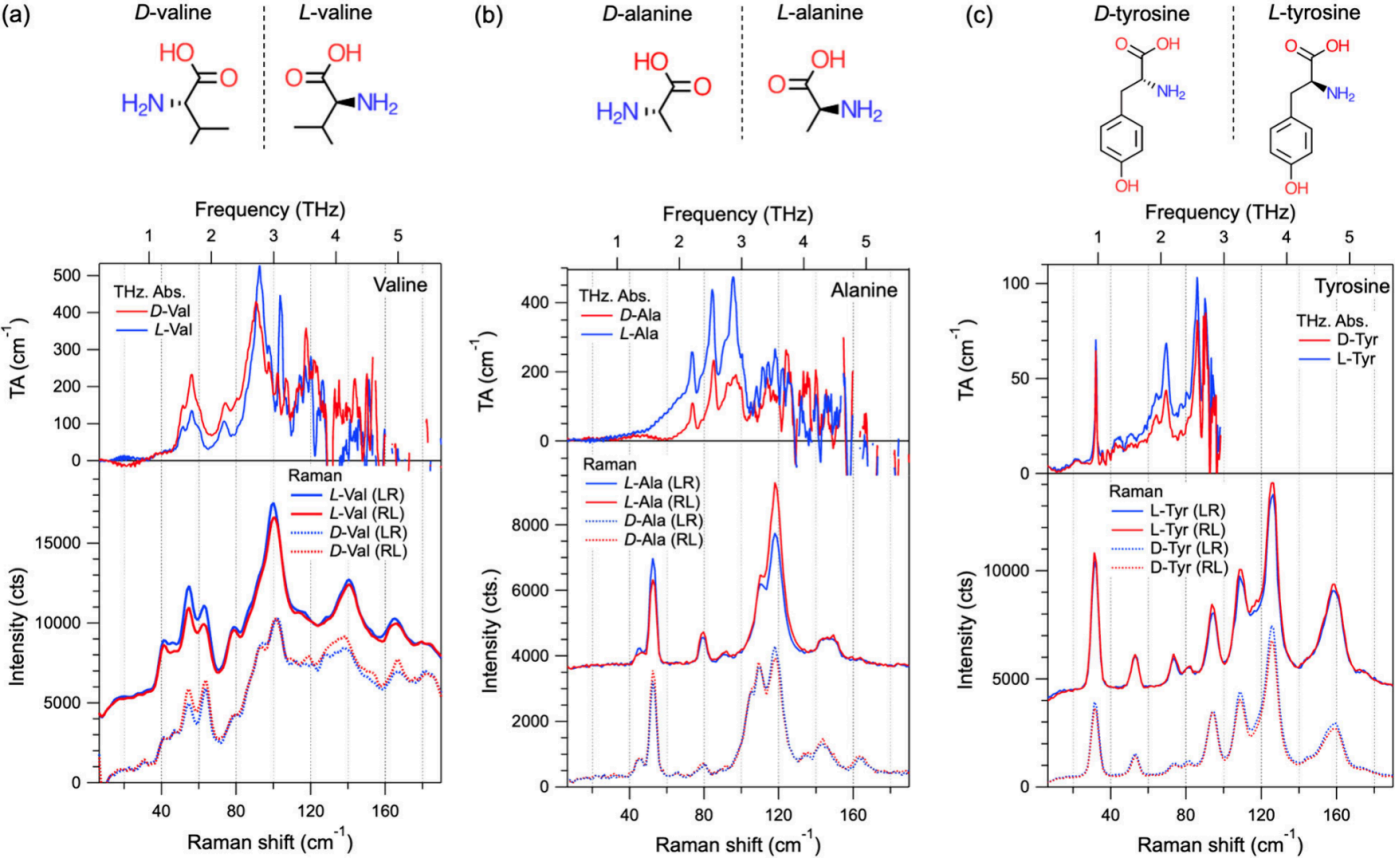
Circularly polarized TA and low-frequency Raman spectra with frequencies corresponding to the top and bottom axes, respectively: (a) *D*- and *L*-Val, (b) *D*- and *L*-Ala, and (c) *D*- and *L*-Tyr crystals. The molecular structures of the three AA enantiomers are shown in the schematics on top.

The TA and TCD measurements were conducted with the help of a custom home-built THz time domain polarimetry setup described in previous studies in refs 6 and 28. The ROA measurements were conducted with RCP or LCP laser excitation (785 nm), achieved by manually inserting a half waveplate and a quarter waveplate prior to the objective lens in our micro-Raman setup. The quarter waveplates are fixed in place and rotated at 45° to transmit RCP or LCP light. The backscattered light is directed through the same waveplates and through a polarizing beamsplitter before entering the spectrometer (a schematic of our optical layout is shown in Figure S1). The scattered light is thus polarized parallel or perpendicular, giving copolarized and cross-circularly polarized configurations (RR/LL and RL/LR, respectively). We note that traditional ROA measurements are conducted by continuously modulating the circular polarization of incident or scattered light (or both). Our spectroscopic setup does not use modulators and is based on the continuous accumulation of spectra for one and the other handedness of the incident light. The setup was validated by collecting cross-circularly polarized ROA spectra from (+)- and (−)-α-pinene (Figure S2). Furthermore, cross-circularly polarized spectra were collected from known standards (single-crystalline silicon and sapphire wafers; Figure S3), and we obtain a detection sensitivity of around 6%.

The TA spectra were collected from concentrated mineral oil slurries containing 50 wt % recrystallized amino acids. For both *D*- and *L*-enantiomers of valine, alanine, and tyrosine, the TA spectra (0.2–5.7 THz) display several sharp absorption peaks between 0.8 and 4 THz, as shown in Figure 1a–c, with frequency ranges indicated as the top axes. These results are consistent with previous far-IR spectra reported for *L*-Val, *L*-Ala, and *L*-Tyr.^30^ Circularly polarized Raman spectra in the low-frequency region were obtained from the AA crystals deposited directly from solution onto silicon substrates and measured under cross-circularly polarized conditions with RL and LR configurations, as displayed in Figure 1. Similar to the TA spectra, the Raman spectra exhibit multiple peaks between 10 and 200 cm^–1^, with peaks from both enantiomers appearing at the same frequencies. These findings are in agreement with previously published low-frequency Raman data on amino acids.^31–33^ Notably, the correspondence between the TA and Raman spectra from valine is strong: below 100 cm^–1^ (3 THz), the two most intense Raman modes at 55 and 63 cm^–1^ align well with the two most intense peaks in the TA spectra at 1.48 and 1.75 THz (Figure 1a), respectively. The strong and sharp TA peak in tyrosine around 0.96 THz can also be observed as an intense Raman mode around 32 cm^–1^ (Figure 1c).

We also observed expected differences between the TA and Raman spectra in the number of peaks and their frequencies, attributed to the fundamentally different selection rules between the two techniques. Similar to conventional IR spectroscopy, the molecular vibrations in TA spectroscopy must cause a net change in their dipole moment. On the contrary, Raman-active vibrations result from a change in the polarizability of a molecule.^29^ These differences between the two techniques, related to the intrinsic symmetries of the crystals and vibrational states, make them complementary. Depending on how the PAM changes the electronic structure of the molecules and the crystal overall, chiral phonon modes may be either Raman- or IR/TA-active or both. The differences between the spectra obtained from the two techniques can be seen more starkly in *D*- and *L*-Ala (Figure 1b), where the most intense peaks in the Raman spectra appear around 48 and 113 cm^–1^ (1.4 and 3.4 THz), respectively, whereas the most intense features in the TA spectra lie between the two frequencies (2.5–3 THz). These peaks between 2.5 and 3 THz do appear as weak features in the Raman spectra. Their appearance can be potentially attributed to differences between the TA and Raman measurements in terms of the probed spot sizes, measurement configurations (i.e., transmission vs backscattering in the TA and Raman measurements, respectively), sample thicknesses, and selection rules. Note that Raman spectra can also access frequencies above 3 THz (100 cm^–1^), allowing for the observation of more extended range structural vibrational modes in the low-frequency range, which is not a methodical restriction but rather a limitation of the currently available experimental setup for TCD.

The origin of the low-frequency peaks in the TA and Raman spectra can be attributed to long-range rotary motions of the chemical groups in the structure of the AA enantiomers. As mentioned above, the *P*2_1_ and *P*2_1_2_1_2_1_ space groups contain 1 and 3 screw axes, respectively, that produce chiral patterns within the AA unit cells. This results in nitrogen atoms of the amino groups (−NH_2_) and N–H···O hydrogen bonds forming helices within each cell. To show the effects of the structural chirality on the vibrational modes, we present TCD and ROA spectra from valine, alanine, and tyrosine in Figure 2. *D*- and *L*-Val exhibit classical bisignate shapes between 1 and 2 THz in their TCD spectra (top panel, Figure 2a). Likewise, *D* and *L*-Tyr exhibit bisignate peaks around 1 THz in their TCD spectra (top panel, Figure 2c). Although the corresponding features in alanine (Figure 2b) are less ideal compared to the common CD spectra in the visible range, the very fact that one can resolve multiple bands with opposite PAM among the many (156–288) closely spaced modes and their superpositions is conceptually and methodologically significant.

**Figure 2:**
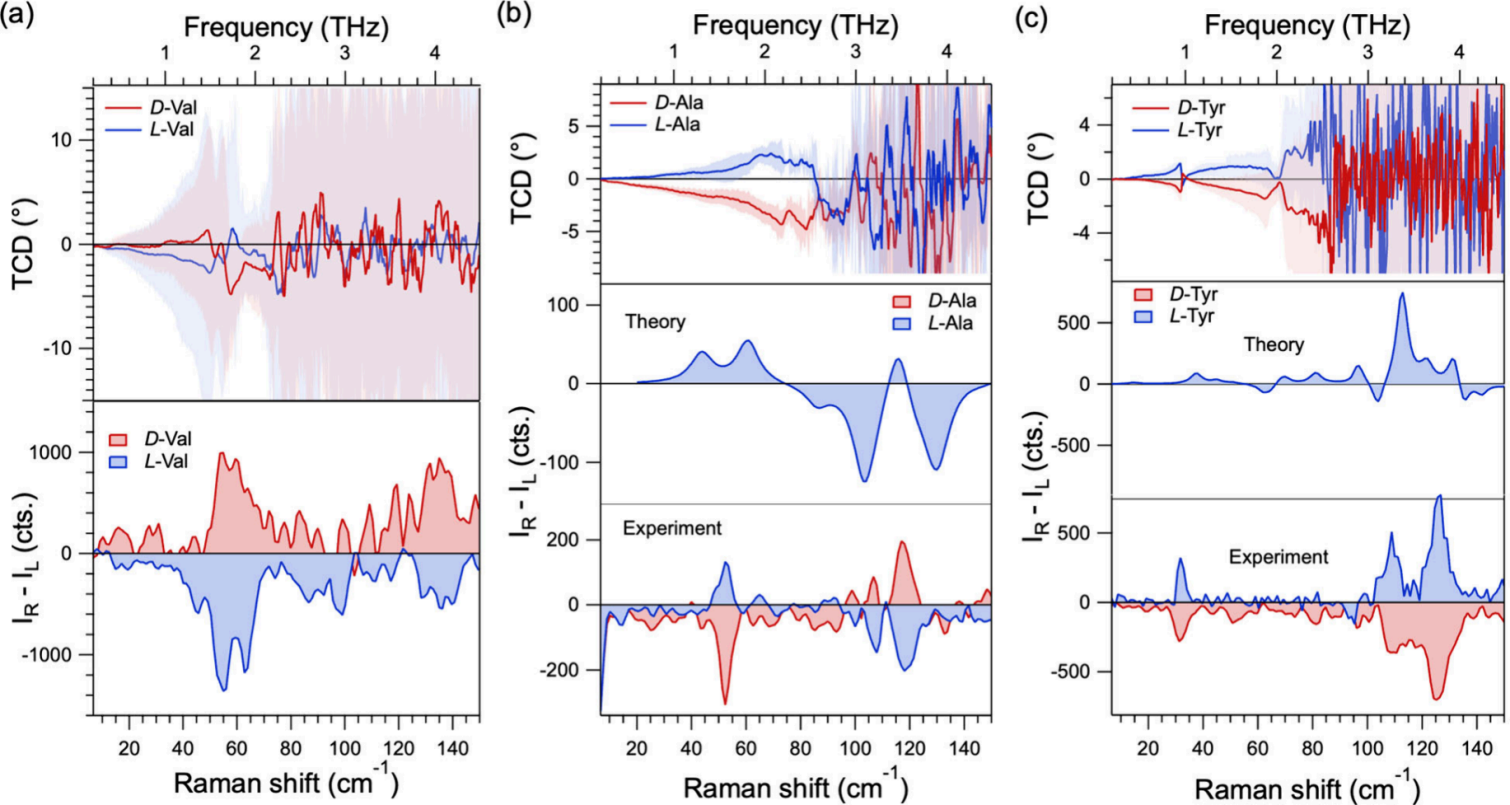
TCD and ROA spectra with corresponding frequencies on the top and bottom axes, respectively, from *D*- and *L*-enantiomers of (a) valine, (b) alanine, and (c) tyrosine. The ROA spectra for *L*-Ala and *L*-Tyr calculated by DFT are also plotted in the middle panels of parts b and c.

Similar to the TCD spectra, the ROA spectra (*I*_RL_ – *I*_LR_, written as *I*_R_ – *I*_L_ for brevity) for all three AAs exhibit opposite mode intensities for RCP and LCP excitation and are consistent with their assignment as chiral phonons. We see these peaks in two frequency regions, one between 40 and 60 cm^–1^ and the second one between 100 and 140 cm^–1^ (bottom panels in Figure 2a–c for valine, alanine, and tyrosine, respectively). The match between the TCD and ROA spectra is particularly good for valine and tyrosine. The ROA peaks in valine appear around 60 cm^–1^, matching well with the bisignate peaks in the TCD spectra around 1.6 THz (Figure 2a). Similarly, tyrosine exhibits a strong and clear chiral phonon signature at the same frequency (32 cm^–1^, or 0.96 THz) in both the TCD and ROA spectra (Figure 2c).

Bisignate ROA peaks can also be seen in the fingerprint regions of the spectra from valine, alanine, and tyrosine (Figure S4) and agree well with previous reports.^25,34^ Other than the three amino acids shown in Figure 2, we also observe ROA peaks in the low-frequency range from enantiomers of glutamine and proline (Figure S5). Both glutamine and proline display greater differences in the TCD and ROA spectra. While the space group for both amino acids is orthorhombic *P*2_1_2_1_2_1_, the organization of dipoles in these lattices is symmetrically different. In the case of *L*-proline, the highly polar amino groups point in the same direction, while for *L*-glutamine, the amino groups display antiparallel alignment, which leads to the large differences in the TCD and ROA spectra as required by the selection rules.

To gain further insights into the origin of the low-frequency Raman modes, we calculated the Raman and ROA spectra of *L*-Ala and *L*-Tyr using density functional theory (DFT; see the Methods section). The theoretical Raman spectra from both amino acids match well with the measured spectrum across both the low-frequency (Figure 2b,c) and fingerprint (Figures S6 and S7, for alanine and tyrosine, respectively) regions in terms of frequencies and relative intensities. The Raman mode calculations for alanine were performed by considering isotropic (polycrystalline) crystals as well as crystals along the *a*, *b* and *c* crystal axes. The calculation for *L*-Ala reveals four Raman modes in the low-frequency region, at 43, 60, 103, and 130 cm^–1^, which are only slightly shifted compared to our experimentally observed peaks at 44, 52, 80, 110, and 118 cm^–1^. Minor discrepancies in frequencies can be attributed to DFT error and anharmonic effects. The lowest frequency peak in the calculated spectrum for *L*-Tyr occurs at 37 cm^–1^, close to the experimentally observed Raman peak around 32 cm^–1^. Importantly, the calculated ROA spectra for *L*-Ala and *L*-Tyr (middle panels in Figure 2b,c) match the experimental spectra very well. Both sets of spectra exhibit positive and negative peaks in the 30–60 cm^–1^ and 100–140 cm^–1^ ranges, respectively, similar to the measured ROA spectra (bottom panels in Figure 2b,c). Note that while we show the calculated ROA for a polycrystal of *L*-Ala in Figure 2b, the spectra along each of the crystallographic axes exhibit ROA peaks with similar signs but varying intensities. All spectra correlate well with the measured ROA spectrum (Figure S8).

For alanine, the calculated Raman modes at 43, 60, 103, and 130 cm^–1^ correspond to twisting and rotary motions that are in opposing directions for the two enantiomers and are characteristic of chiral phonons. This is similar for the calculated modes for tyrosine at 37, 97, 113, 133, and 175 cm^–1^. The eigenvectors for two of the modes at 60 and 130 cm^–1^ for alanine and the modes at 37 and 133 cm^–1^ for tyrosine are shown in parts a and b of Figure 3, respectively. The eigenvectors for the other calculated modes for alanine and tyrosine are shown in Figures S9 and S10, including animations of all mode vibrations. Note that the schematics in Figures 3, S9, and S10 show the zwitterionic forms of *L*-Ala and *L*-Tyr. The modes at 43 and 60 cm^–1^ primarily involve shearing motions of the molecules within the alanine unit cell, accompanied by a small amount of twisting motion. The 103 and 130 cm^–1^ modes primarily involve twisting of the carboxylate and methyl groups in opposite directions. For tyrosine, all of the low-frequency modes involve twisting motions of the molecules. We also calculated the TA spectrum for *L*-Ala (Figure S11), which shows a good match with the experimentally observed peaks at 1.4 and 1.8 THz. The calculations are consistent with previous work that showed that the 1.2–1.4 THz peak in the TA spectrum from *L*-glutamine involves the twisting of the carboxylate groups in both the main and side chains.^10^ Collectively, our analysis indicates that the chiral phonons in the amino acids correspond to long-range twisting motions of the constituent functional groups.

**Figure 3:**
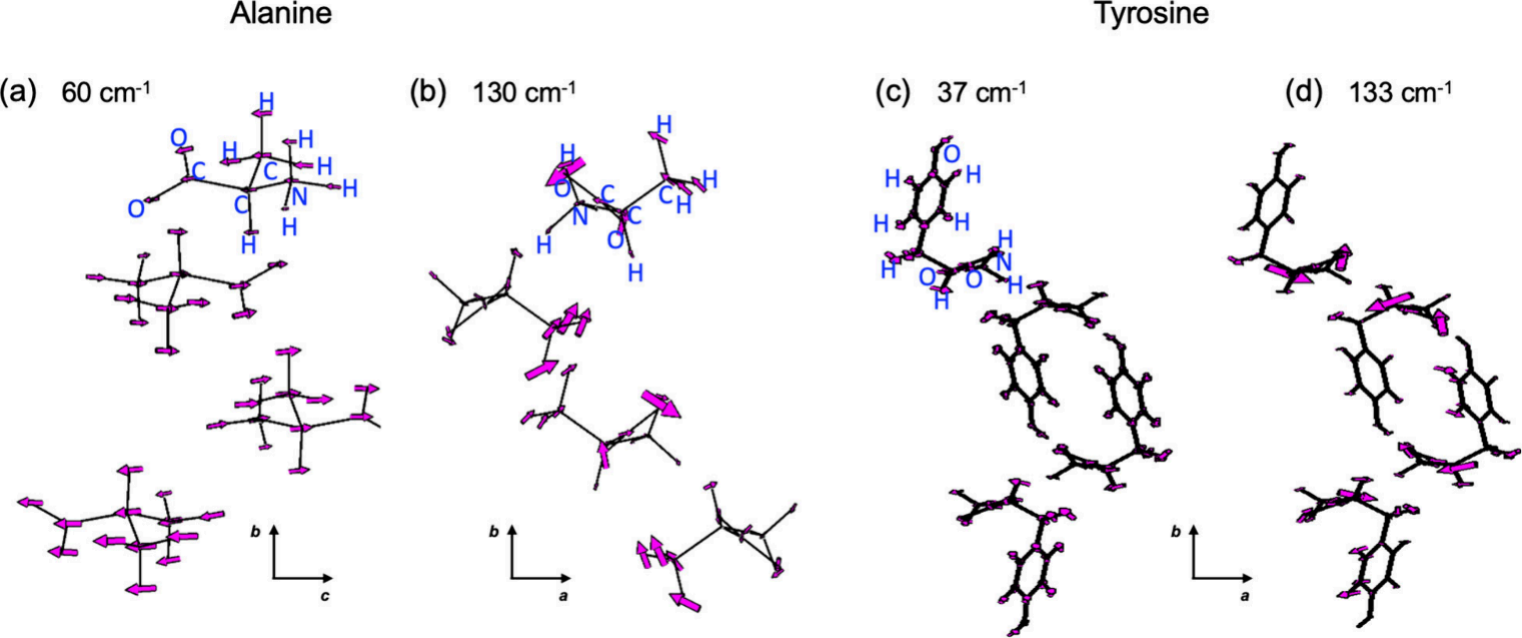
Schematics of the eigenvectors for two calculated Raman modes at (a) 60 and (b) 130 cm^–1^ for *L*-Ala and Raman modes at (c) 37 and (d) 133 cm^–1^ for *L*-Tyr. The atoms are labeled for the top molecule in the alanine and tyrosine unit cells.

We note that while we observed differences in intensities in the chiral phonon modes with RCP or LCP excitation, we do not see any splitting in peak frequencies. One reason could be the presence of several overlapping vibrational modes with closely spaced frequencies, as revealed by the calculations (Figure S5). Another reason could be that the splitting is present and finite but is small enough that it is below our instrument resolution of 1 cm^–1^. Indeed, such a small spitting and opposite angular momenta were recently calculated for the achiral 2D material AgCrP_2_Se_6_ and chiral hybrid organic–inorganic perovskites,^35,36^ which did not exhibit any splitting of frequencies in room temperature Raman spectra, but nonetheless exhibited ROA for several low-energy Raman modes. ROA spectra with negligible or very small frequency splitting have also been reported for other achiral materials such as TaS_2_, ReS_2_, and ReSe_2_ and chiral materials like NaClO_3_.^37–42^

In summary, we have presented room-temperature TA, TCD, Raman, and ROA spectra from AA crystals, showing multiple complementary peaks in the frequency region below 150 cm^–1^ or 4.5 THz, indicative of the chiral phononic states of crystals with a large number of atoms. The ROA data show peaks arising from chiral vibrational modes, with intensities greater than those in the fingerprint region. Computations of the Raman and ROA spectra for *L*-Ala and *L*-Tyr revealed several low-frequency Raman-active modes corresponding to shearing and twisting of the alanine and tyrosine molecules within their unit cell as well as twisting of the constituent functional groups. Overall, our study highlights the multiplicity of chiral phonon states in crystals of bioorganic molecules that can be identified by both TCD and ROA. The strong influence of structural chirality on the interaction between chiral phonons and circularly polarized light demonstrates the complementary nature of low-frequency circularly polarized Raman spectroscopy and TCD for identifying low-energy chiral phonons in organic materials.

## Methods

### TA and TCD Measurements

For the TA and TCD measurements, we used THz time-domain polarimetry based on a 1550 nm fiber-coupled femtosecond laser (>500 mW, 100 MHz) with a TERA15-TX-FC antenna (Menlo Systems) as the THz emitter. For detection, a delayed probe beam was used with a TERA15-RX-FC antenna (Menlo Systems), and the voltage signal was amplified. The system employed four TPX lenses (*f* = 50 mm) to collimate the THz beam and focus it at the sample position, producing a focal spot of ∼500 μm in diameter at 1 THz, with a depth of focus of roughly 1 mm. Three THz linear polarizers (Microtech, G30-s) were used: the first and third, placed directly in front of the THz emitter and detector, ensured linearly polarized light, while the second polarizer was rotated to measure TCD and TORD. For sample preparation, all AA samples were purchased from Sigma-Aldrich and ground into small crystals using a mortar and pestle. The powders were then mixed with mineral oil to form a slurry. The sample slurries were spread onto a quartz slide and sandwiched with another quartz slide. The slurry thickness was controlled using a spacer; in this case, we used 3M double-sided tape.

### Circularly Polarized Raman Spectroscopy

Room temperature circularly polarized Raman spectra were collected with a Renishaw inVia Raman microscope. Our instrument is outfitted with a low-frequency module (Coherent/Ondax THz Raman probe) that uses fiber optics to couple a 785 nm laser into the objective lens for excitation and to direct the scattered light into the inVia spectrometer. The low-frequency module is equipped with a notch filter that provides a high degree of laser light rejection, enabling the measurement of Raman peaks down to 10 cm^–1^. The optical layout for achieving circular polarization is shown in Figure S1. *L*- and *D*-amino acids were purchased from Sigma-Aldrich at >98% purity and dissolved in doubly deionized water to yield concentrations of 10 mg/mL. A total of 2 μL of each AA enantiomer was spotted onto a single-sided polished silicon wafer and allowed to air-dry at room temperature for 2 h. All spectra were collected by focusing the excitation laser on the AA crystals through a 50× objective lens. The laser power used for the spectral collection was 1.8 mW, and spectra were collected with a 10 s exposure time and up to 60 accumulations. At least three spectra were collected from different spots and averaged to obtain the LR and RL spectra.

### Single-Crystal X-ray Diffraction

X-ray diffraction measurements were performed with a Rigaku KtaLAB synergy-I single-crystal diffractometer with Cu Kα radiation (λ = 1.5406 Å). Refinements were completed with onboard Rigaku *CrysAlis Pro* software using *ShelX* ^43^ and are presented in Tables S1 and S2 for *D*-Val and *L*-Ala, respectively.

### Raman and ROA Calculations for Alanine

The crystal cell and geometry of a *L*-alanine crystal were first optimized by energy minimization using the *CASTEP* software.^44^ The X-ray structure was used as the initial geometry (number 278466 in the Cambridge Crystallographic Data Centre), and the PBE functional was used with a 700 eV basis set cutoff, which provided lattice cell parameters (5,88, 11.86, and 5.73 Å) comparable with the lattice parameters obtained experimentally (5.94, 12.26, and 5.79 Å). The harmonic force field (dynamic matrix) and zero-phonon eigenvectors were obtained at the same level as the optimization. To get polarizability derivatives needed for Raman and ROA,^45^ a cluster of alanine molecule and nearest neighbors was partially optimized in the vibrational normal mode coordinates^46^ and the *Gaussian* program^47^ was used, applying the B3LYP/6-311++G**/PCM method. The polarizability derivatives were then transferred on the lattice cell geometry using the Cartesian-coordinate-based tensor transfer,^48^ and backscattered Raman and ROA intensities for the scattered circular polarization (SCP) were generated. Smooth spectra were obtained by a convolution of the intensities with Lorentzian functions using a bandwidth of 3 or 10 cm^–1^.

### Raman and ROA Calculations for Tyrosine

The cell X-ray geometry of a *L*-tyrosine crystal was downloaded from the CDCC database (https://www.ccdc.cam.ac.uk/), deposition number 1208549 (crystal dimensions 6.913 Å × 21.116 Å × 5.829 Å). The geometry was first optimized by energy minimization using the *CASTEP* software,^44^ with the PBE functional and 900 eV basis set cutoff, which provided lattice cell parameters 6.836, 21.098, and 5.863 Å. The harmonic force field (dynamic matrix), zero-phonon eigenvectors, and dipole and polarizability derivatives were obtained at the same level as the optimization. The polarizability derivatives were alternatively obtained by the *Gaussian* program^47^ for tyrosine pairs. This enabled us to calculate also intrinsic magnetic dipole and electric quadrupole polarizabilities needed for ROA (tensors G and A; cf. ref 45). Seven pairs (dimers) were chosen so that all nearest-neighbor molecular interactions within the crystal cell and for molecules in and outside the cell were included. The dimers were partially optimized in the vibrational normal mode coordinates^46^ and the polarizability derivatives calculated at the B3LYP/6-311++G**/PCM level were then transferred on the lattice cell geometry using the Cartesian-coordinate-based tensor transfer^48^ to generate backscattered Raman and ROA intensities for the SCP configuration. Smooth spectra were obtained by a convolution of the intensities with Lorentzian functions using a bandwidth of 5 or 10 cm^–1^. Using the distributed origin gauge^49^ local parts of the ROA tensor derivatives were combined with the polarizability derivatives from *CASTEP*.

## Supplementary Material



## Notes

Single-crystal X-ray diffraction data from valine and alanine, optical layout of our Raman setup, additional Raman and TA spectra from the amino acids, and eigenvectors for Raman-active chiral phonon modes in alanine and tyrosine (PDF)
