# Supplementary material for: Raman and Terahertz Spectroscopy of Low-Frequency Chiral Phonons in Amino Acids
Source: Nano Lett. 2026 Apr 20;26(17):5713–9. doi: 10.1021/acs.nanolett.6c00060 (PMC13154365; doi:10.1021/acs.nanolett.6c00060)
Supplement: Supplementary file 1 [file nl6c00060_si_001.pdf]

Supporting Information

Raman and Terahertz Spectroscopy of Low-Frequency Chiral Phonons in Amino Acids

Rahul Rao,*,[†] Wonjin Choi,*,[‡] Joseph M. Slocik,[†,¶] Thuc T. Mai,[†,¶] Michael A. Susner,[†] Kelsey A. Collins,[†,^] Michael J. Newburger,[†] Petr Bouř,[§]* and Nicholas A. Kotov*,[‖,⊥,#,@]

[†]Materials and Manufacturing Directorate, Air Force Research Laboratory, Wright-Patterson AFB, Ohio 45433, United States

[‡]Physical and Life Sciences, Lawrence Livermore National Laboratory, Livermore, California 94550, United States

[¶]BlueHalo, an AV company, Dayton, Ohio 45433, United States

[^]Core4ce, Dayton, OH 45422, United States

[§]Institute of Organic Chemistry and Biochemistry, Flemingovo nám. 2, 16610 Prague, Czech Republic

[‖]Department of Chemical Engineering, University of Michigan, Ann Arbor, Michigan 48109, United States

[⊥]Biointerfaces Institute, University of Michigan, Ann Arbor, Michigan 48109, United States

[#]Department of Materials Science and Engineering, University of Michigan, Ann Arbor, Michigan 48109, United States

[@]Center for Complex Particle Systems, University of Michigan, Ann Arbor, Michigan 48109, United States

E-mail: rahul.rao.2@us.af.mil; choi21@llnl.gov; petr.bour@uochb.cas.cz; kotov@umich.edu

Table S1. Single crystal X-ray diffraction refinement for *D*-Val

Lattice constants	Atom	Site	Wycoff pos.	Atomic Coordinates			Occ. f.	$U_{eq/iso}$ (Å^2)*
				X	Y	Z		
a= 9.6757(2) Å	O	O3	2a	0.1290(2)	0.6072(6)	0.6009(2)	1	0.0474(7)
b= 5.27530(14) Å	O	O1	2a	0.6453(2)	0.2087(6)	0.6038(2)	1	0.0480(7)
c= 12.0398(3) Å	O	O4	2a	0.2081(2)	0.9744(5)	0.6629(2)	1	0.0501(7)
$\alpha = \gamma$ = 90°	O	O2	2a	0.7263(3)	0.5977(6)	0.6313(3)	1	0.0555(8)
β = 90.758(2)°	N	N2	2a	0.3768(3)	0.3922(6)	0.5900(3)	1	0.0406(7)
$P2_1$ (4)	H	H2A	2a	0.3574	0.4228	0.5188	1	0.0490
	H	H2B	2a	0.316945	0.279975	0.616372	1	0.049
	H	H2C	2a	0.4622	0.3304	0.5964	1	0.0490
	N	N1	2a	0.9110(3)	0.0262(7)	0.6250(3)	1	0.0472(9)
	H	H	2a	0.9057	0.0640	0.5530	1	0.0570
	H	HA	2a	0.8568	-0.1060	0.6390	1	0.0570
	H	HB	2a	0.9980	-0.0121	0.6432	1	0.0570
	C	C7	2a	0.2230(3)	0.7489(8)	0.6369(3)	1	0.0400(8)
	C	C2	2a	0.7356(3)	0.3663(8)	0.6359(3)	1	0.0402(8)
	C	C1	2a	0.8652(3)	0.2481(8)	0.6915(3)	1	0.0409(8)
	H	HC	2a	0.9393	0.3747	0.6929	1	0.0490
	C	C6	2a	0.3669(3)	0.6322(7)	0.6544(3)	1	0.0405(8)
	H	H6	2a	0.4352	0.7511	0.6252	1	0.0490
	C	C8	2a	0.4026(4)	0.5830(9)	0.7762(3)	1	0.0471(9)
	H	H8	2a	0.4769	0.4568	0.7784	1	0.0570
	C	C3	2a	0.8353(3)	0.1671(9)	0.8108(3)	1	0.0467(10)
	H	HD	2a	0.7615	0.0398	0.8077	1	0.0560
	C	C9	2a	0.4575(5)	0.8222(11)	0.8328(4)	1	0.0658(13)
	H	H9A	2a	0.5330	0.8893	0.7910	1	0.0990
	H	H9B	2a	0.4890	0.7816	0.9066	1	0.0990
	H	H9C	2a	0.3851	0.9462	0.8364	1	0.0990
	C	C10	2a	0.2810(4)	0.4734(10)	0.8406(4)	1	0.0581(11)
	H	H10A	2a	0.2091	0.5982	0.8453	1	0.0870
	H	H10B	2a	0.3115	0.4273	0.9140	1	0.0870
	H	H10C	2a	0.2461	0.3260	0.8027	1	0.0870
	C	C4	2a	0.9605(4)	0.0450(11)	0.8672(4)	1	0.0635(13)
	H	HE	2a	0.9879	-0.1018	0.8258	1	0.0950
	H	HF	2a	0.9371	-0.0044	0.9413	1	0.0950
	H	HG	2a	1.0353	0.1644	0.8699	1	0.0950
	C	C5	2a	0.7841(6)	0.3869(13)	0.8803(4)	1	0.0740(15)

	H	HH	2a	0.8533	0.5172	0.8830	1	0.1110
	H	HI	2a	0.7659	0.3289	0.9543	1	0.1110
	H	HJ	2a	0.7007	0.4537	0.8477	1	0.1110

Reliability Factors	R_1	wR_2	R_{int}	GooF
	0.0562	0.1575	0.0462	1.078

*all H atoms were refined isotropically

Atom	U_{11} (Å^2)	U_{22} (Å^2)	U_{33} (Å^2)	U_{23} (Å^2)	U_{13} (Å^2)	U_{12} (Å^2)
O3	0.0293(11)	0.0529(18)	0.0601(15)	-0.0029(14)	0.0022(9)	0.0013(11)
O1	0.0318(12)	0.0473(17)	0.0648(16)	-0.0073(13)	-0.0034(10)	0.0055(11)
O4	0.0395(13)	0.0369(17)	0.0739(17)	-0.0009(14)	0.0017(11)	0.0066(12)
O2	0.0507(15)	0.0385(18)	0.0771(19)	0.0018(15)	-0.0036(13)	0.0085(13)
N2	0.0289(13)	0.0388(18)	0.0542(17)	0.0001(14)	0.0026(11)	0.0067(13)
N1	0.0339(14)	0.051(2)	0.0572(18)	0.0053(16)	0.0022(12)	0.0096(14)
C7	0.0308(15)	0.037(2)	0.052(2)	0.0052(17)	0.0065(13)	0.0007(15)
C2	0.0332(16)	0.040(2)	0.0474(19)	-0.0030(17)	0.0030(13)	0.0040(16)
C1	0.0284(15)	0.040(2)	0.055(2)	-0.0001(17)	0.0003(13)	-0.0002(14)
C6	0.0279(15)	0.036(2)	0.058(2)	-0.0038(17)	0.0042(13)	-0.0028(15)
C8	0.0369(17)	0.046(2)	0.059(2)	-0.0024(18)	-0.0011(14)	0.0042(16)
C3	0.0340(16)	0.051(3)	0.055(2)	0.0019(18)	0.0000(13)	-0.0014(17)
C9	0.056(2)	0.066(3)	0.075(3)	-0.009(3)	-0.009(2)	-0.004(2)
C10	0.059(2)	0.059(3)	0.056(2)	0.002(2)	0.0079(18)	-0.003(2)

Table S2. Single crystal X-ray diffraction refinement for *L*-Ala

Lattice constants	Atom	Site	Wycoff pos.	Atomic Coordinates			$U_{eq/iso}$ (Å^2)*	Occ. f.
				x	y	z		
a=5.7892(2) Å	O	O1	4a	0.23900	0.55390	0.68464	0.0313(4)	1
b=12.2976(3) Å	O	O2	4a	0.37400	0.27250	0.58403	0.0316(4)	1
c=5.9800(2) Å	N1	N1	4a	0.81530	0.34880	0.63787	0.0257(5)	1
$\alpha = \beta = \gamma = 90^o$	H	H1A	4a	0.79800	0.30099	0.56809	0.031	1
	H	H1B	4a	0.79182	0.23228	0.68418	0.031	1
$P2_12_12_1$ (19)	H	H1C	4a	0.96072	0.40327	0.64721	0.031	1
	C	C1	4a	0.39930	0.44240	0.64106	0.0244(5)	1
	C	C2	4a	0.64390	0.52860	0.66102	0.0248(5)	1
	H	H2	4a	0.65786	0.57220	0.73925	0.030	1
	C	C3	4a	0.69630	0.73200	0.59141	0.0321(5)	1
	H	H3A	4a	0.84979	0.78978	0.60987	0.048	1

	H	H3B	4a	0.58015	0.84784	0.60495	0.048	1
	H	H3C	4a	0.69293	0.68954	0.51445	0.048	1
Reliability Factors		R_1	wR_2	R_{int}	GooF			
		0.0334	0.0796	0.0309	1.093			

*all H atoms were refined isotropically

Atom	U_{11} (Å^2)	U_{22} (Å^2)	U_{33} (Å^2)	U_{23} (Å^2)	U_{13} (Å^2)	U_{12} (Å^2)
O1	0.0229(8)	0.0399(8)	0.0312(7)	-0.0051(7)	0.0026(6)	0.0010(8)
O2	0.0276(9)	0.0382(8)	0.0289(7)	-0.0062(6)	-0.0015(6)	-0.0028(7)
N1	0.0208(10)	0.0326(8)	0.0236(8)	-0.0023(7)	-0.0001(7)	-0.0004(7)
C1	0.0244(11)	0.0317(10)	0.0170(8)	0.0020(8)	-0.0002(8)	-0.0009(9)
C2	0.0218(11)	0.0316(10)	0.0209(8)	-0.0026(8)	-0.0009(8)	0.0003(9)
C3	0.0267(13)	0.0352(11)	0.0345(10)	0.0018(10)	0.0000(9)	-0.0022(10)

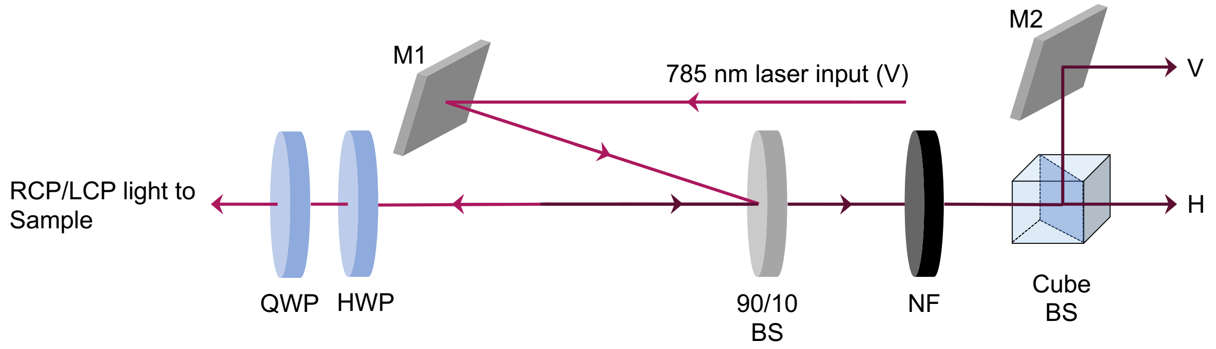

Figure S1. Optical layout showing the configurations for circularly polarized excitation. QWP and HWP are the quarter and half waveplates respectively, M1 and M2 are mirrors, NF is the notch filter and the two beamsplitters are labeled BS. In our configuration, the laser excitation is polarized vertically (laboratory coordinates) and directed onto the sample by mirror M1 and through a 90/10 BS. The vertical polarization can be converted to LCP or RCP by manually placing HWP and/or QWP into the beam path. The backscattered light from the sample passes through the same waveplates, which reverses the circular polarization of the scattered light (e.g. incident LCP is converted to scattered RCP). This light passes through the 90/10 BS and NF and is converted into linearly polarized (vertical, V or horizontal, H) light with a polarizing cube BS at the exit port. The scattered light is then taken into the spectrometer using a polarization maintaining optical fiber. Prior to the circularly polarized measurements, we performed detailed measurements of the polarization state (through the combination of a half waveplate and quarter waveplate) using a polarimeter (Thorlabs PAX1000IR1). The Stokes parameter S_3 of the left and right circular polarizations are -0.995 and 0.996 respectively.

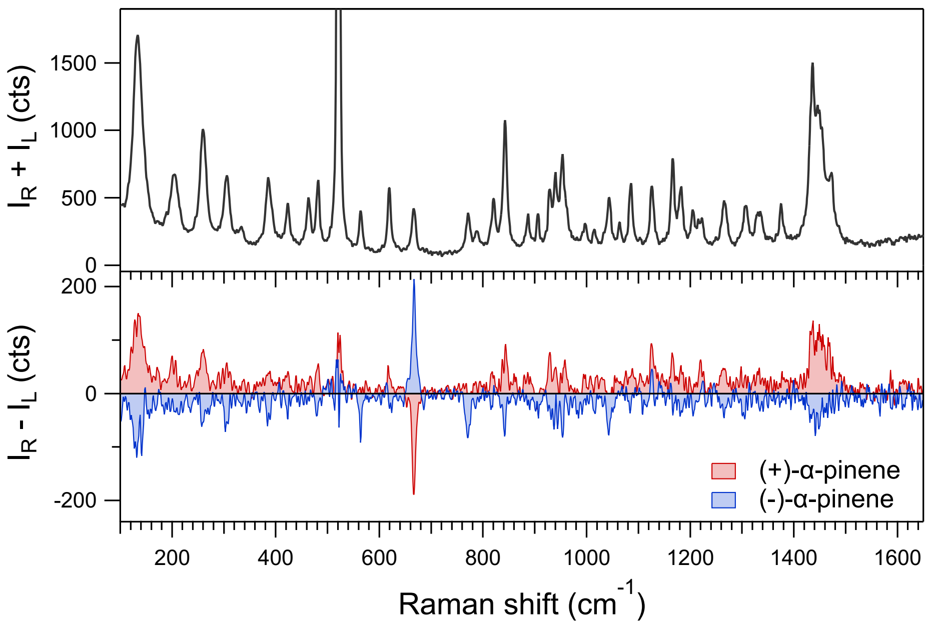

Figure S2. Raman ($I_R + I_L$) and ROA ($I_R - I_L$) from (+)- and (-)-α-Pinene.

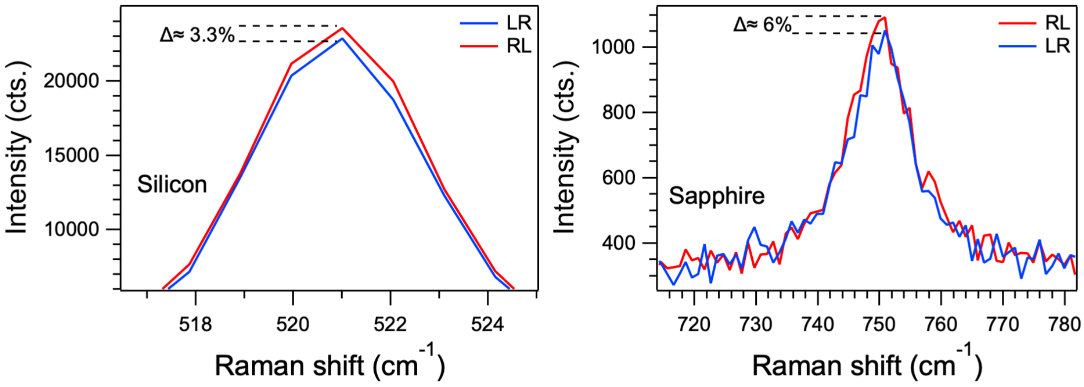

Figure S3. Cross-circularly polarized Raman spectra from silicon (100) (left) and c-cut sapphire wafers (right). The detection sensitivity varies between 3 and 6% and was obtained by peak fitting.

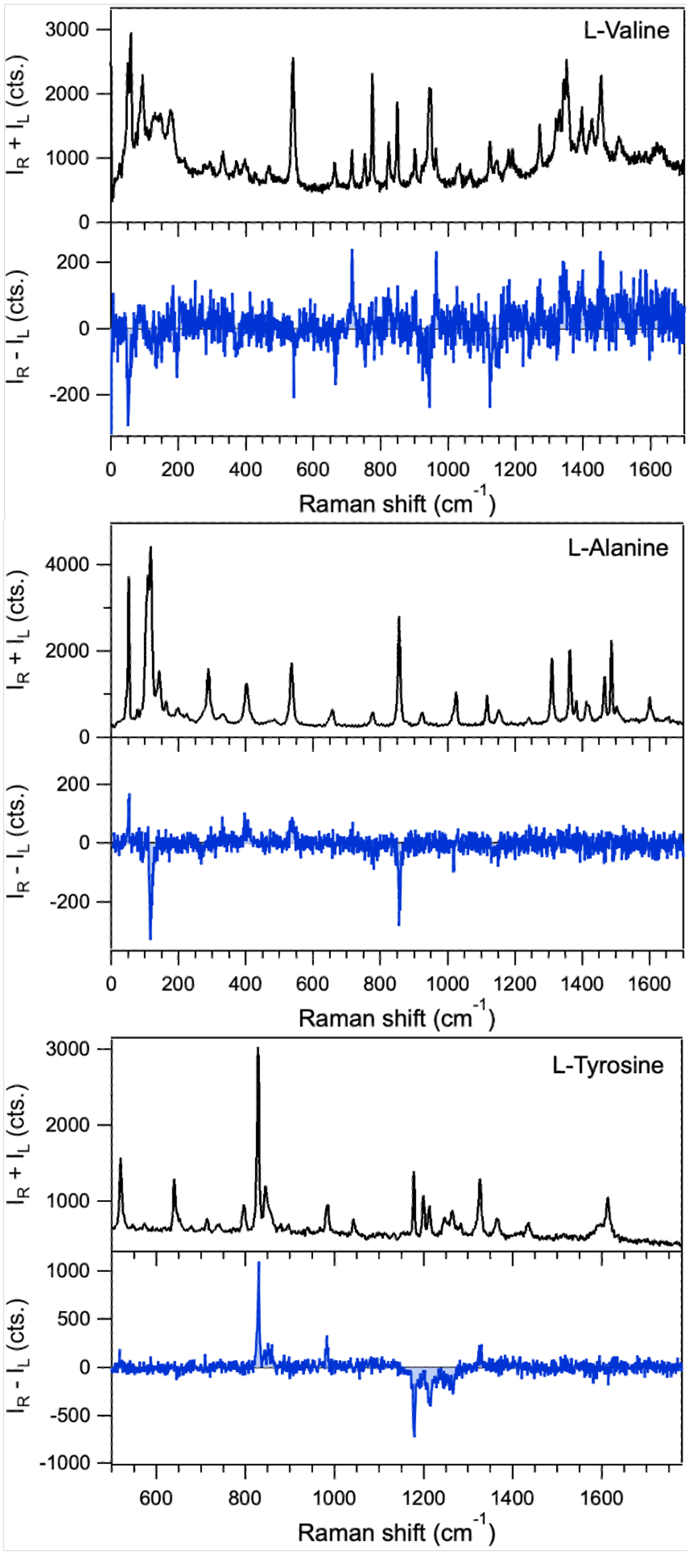

Figure S4. Raman ($I_R + I_L$) and ROA ($I_R – I_L$) in the high-frequency fingerprint regions from *L*-Valine (top), *L*-Ala (middle), and *L*-Tyr (bottom).

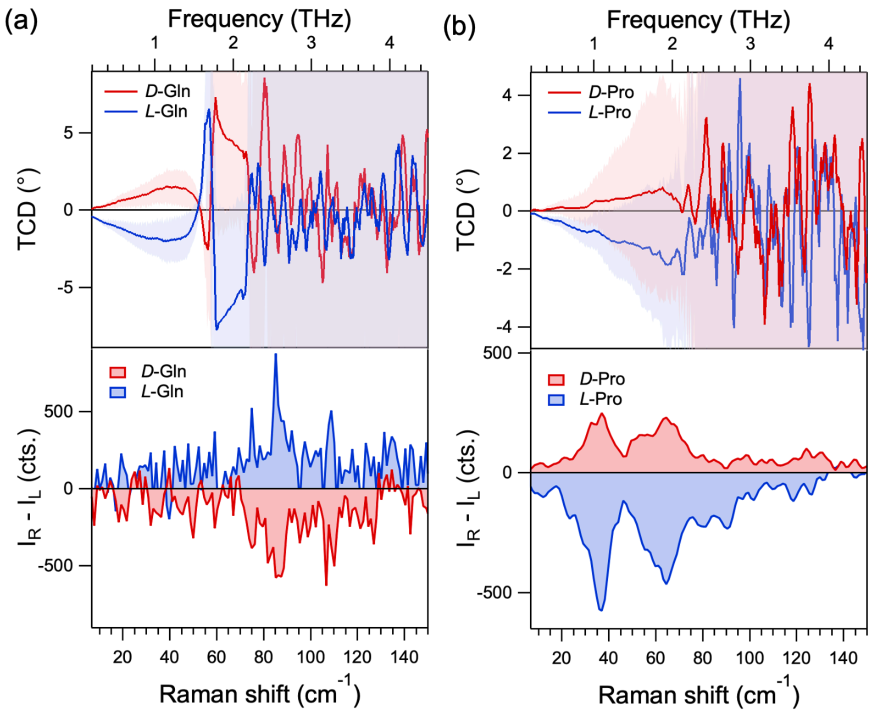

Figure S5. TCD and ROA spectra (frequencies corresponding to the top and bottom axes, respectively) from *D* and *L* enantiomers of (a) glutamine[1] and (b) proline.

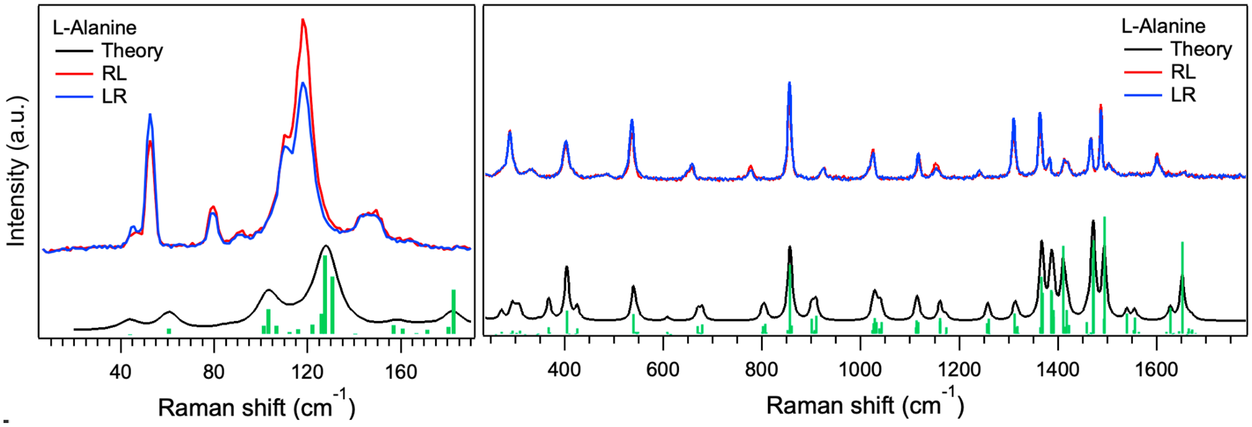

Figure S6. Theoretical and (circularly-polarized) experimental Raman spectra from *L*-Ala in the low-frequency (left) and fingerprint regions (right). The green vertical lines show the line intensities for the calculated modes and the spectra in the two figures have been vertically scaled for clarity.

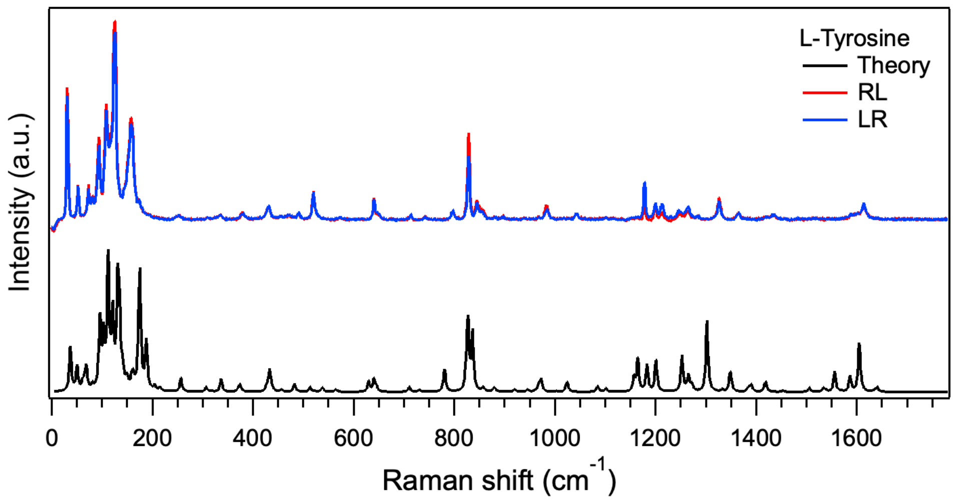

Figure S7. Theoretical and (circularly-polarized) experimental Raman spectra from *L*-Tyr.

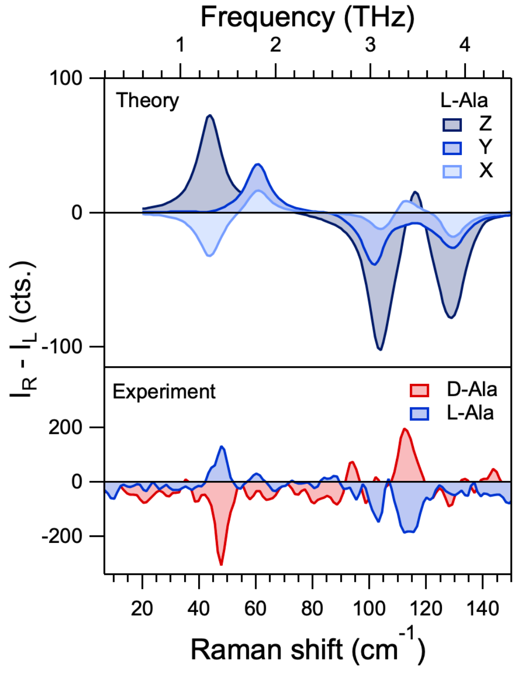

Figure S8. Top - Calculated ROA spectrum from *L*-Ala along the three crystallographic directions, and, Bottom – experimentally measured ROA spectra from *D*- and *L*-Ala. The intensities of the calculated spectra have been multiplied by 100 to match the experimentally observed intensities.

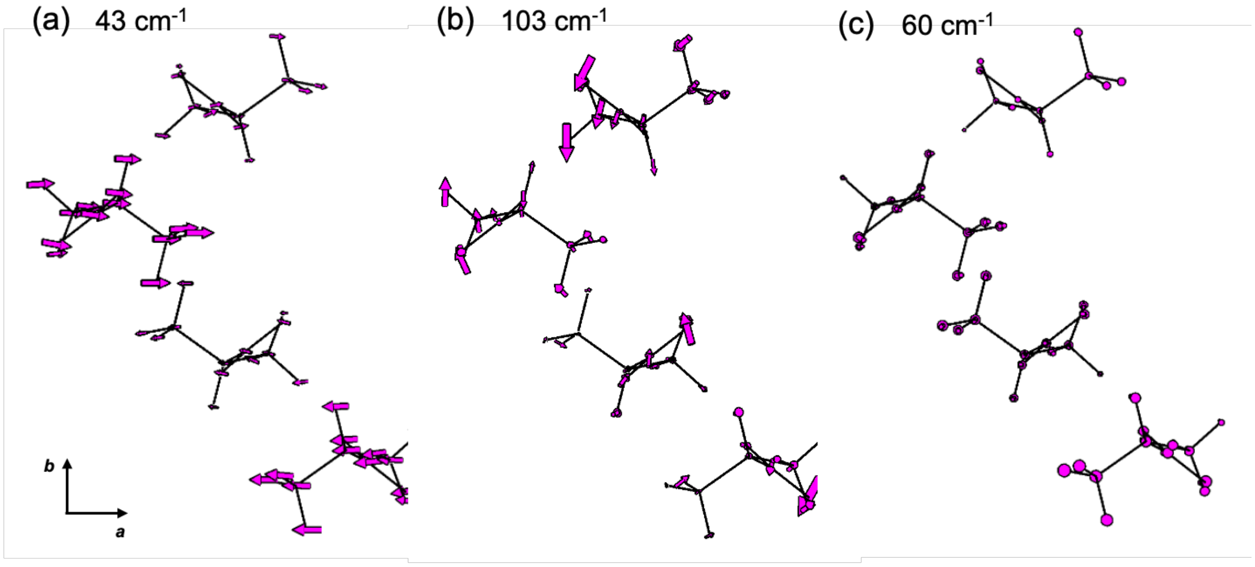

Figure S9. Eigenvectors for the calculated low-frequency Raman modes at (a) 43 cm^{-1}, (b) 103 cm^{-1} and (c) 60 cm^{-1} in *L*-Ala.

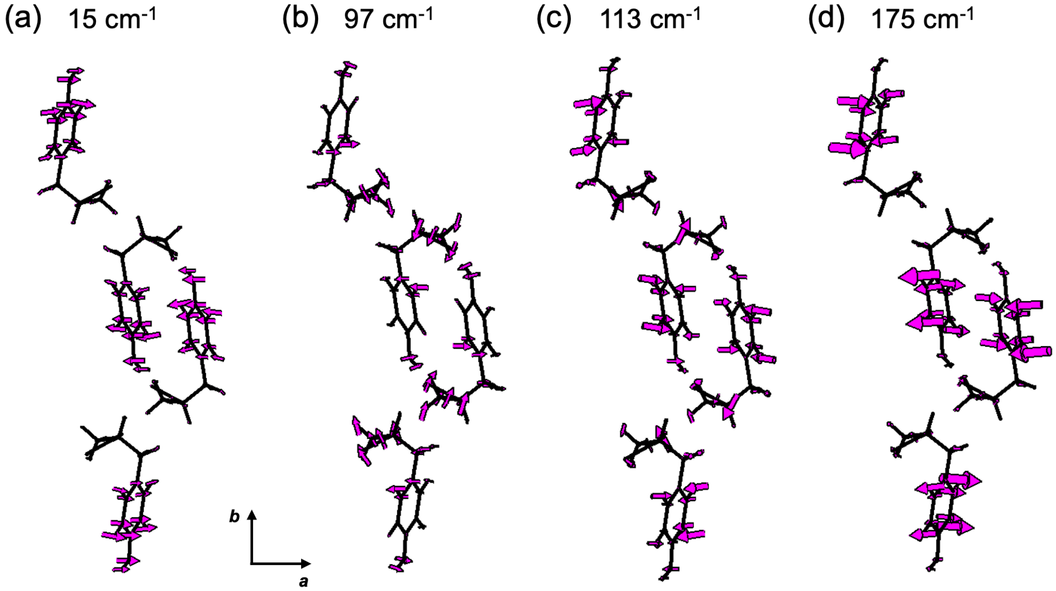

Figure S10. Eigenvectors for the calculated low-frequency Raman modes at (a) 15, (b) 97 cm^{-1}, (c) 113 cm^{-1} and (d) 175 cm^{-1} in *L*-Tyr.

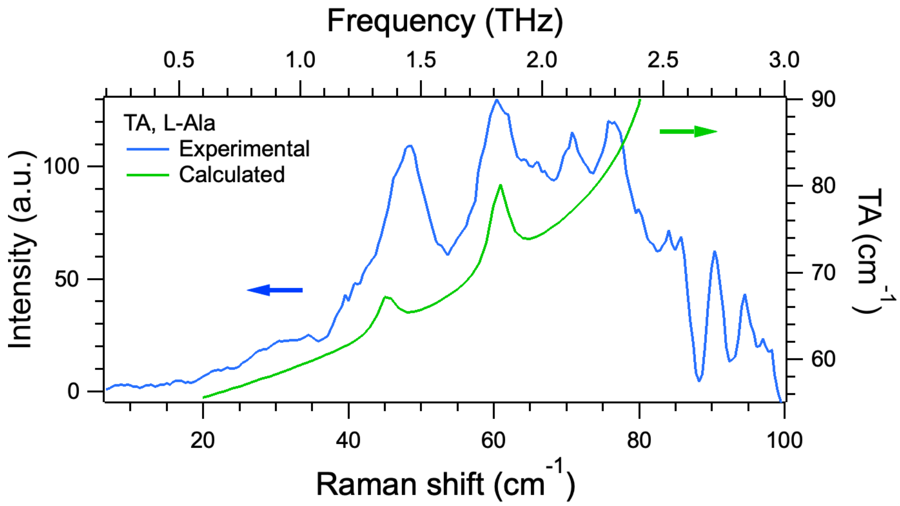

Figure S11. Experimental (left axis) and calculated TA spectra (right axis) from *L*-Ala.

References

(1) Choi, W. J.; Yano, K.; Cha, M.; Colombari, F. M.; Kim, J.-Y.; Wang, Y.; Lee, S. H.; Sun, K.; Kruger, J. M.; de Moura, A. F.; Kotov, N. A. Chiral Phonons in Microcrystals and Nanofibrils of Biomolecules. *Nat. Photonics* **2022**, *16* (5), 366–373. https://doi.org/10.1038/s41566-022-00969-1.
